# Environmental adaptation in stomatal size independent of the effects of genome size

**DOI:** 10.1111/nph.13076

**Published:** 2014-09-30

**Authors:** Gregory J Jordan, Raymond J Carpenter, Anthony Koutoulis, Aina Price, Timothy J Brodribb

**Affiliations:** 1 School of Biological Sciences, University of TasmaniaPrivate Bag 55, Hobart, Tasmania, 7001, Australia; 2 School of Earth and Environmental Sciences, Benham Bldg DX 650 312, University of AdelaideAdelaide, South Australia, 5005, Australia

**Keywords:** adaptation, cell size, chromosome size, CO _2_, genome size, palaeoproxy, Proteaceae, stomata

## Abstract

- Cell sizes are linked across multiple tissues, including stomata, and this variation is closely correlated with genome size. These associations raise the question of whether generic changes in cell size cause suboptimal changes in stomata, requiring subsequent evolution under selection for stomatal size.
- We tested the relationships among guard cell length, genome size and vegetation type using phylogenetically independent analyses on 67 species of the ecologically and structurally diverse family, Proteaceae. We also compared how genome and stomatal sizes varied at ancient (among genera) and more recent (within genus) levels.
- The observed 60-fold range in genome size in Proteaceae largely reflected the mean chromosome size. Compared with variation among genera, genome size varied much less within genera (< 6% of total variance) than stomatal size, implying evolution in stomatal size subsequent to changes in genome size. Open vegetation and closed forest had significantly different relationships between stomatal and genome sizes.
- Ancient changes in genome size clearly influenced stomatal size in Proteaceae, but adaptation to habitat strongly modified the genome–stomatal size relationship. Direct adaptation to the environment in stomatal size argues that new proxies for past concentrations of atmospheric CO_2_ that incorporate stomatal size are superior to older models based solely on stomatal frequency.

## Introduction

Stomata (the microscopic valves that regulate the evaporative loss of water while leaves absorb CO_2_) are critical to the ability of plants to thrive on land. Both the size and abundance of stomata are important because together they determine the maximum capacity of leaves to absorb CO_2_. This intimate link to the uptake of CO_2_ means that stomata are not only pivotal in terrestrial primary productivity, but can also be used (when fossilized) to estimate how atmospheric CO_2_ has changed through time (Royer, 2001; Grein *et al*., 2013). Recent work has shown that the size of stomata is important for whole-plant function because the geometry of stomata combined with constraints on how many stomata can be packed into an area of leaf means that leaves with large stomata tend to have lower maximum capacity to absorb CO_2_ (Franks & Beerling, 2009; Brodribb *et al*., 2013). In addition, there is evidence that, in angiosperms, smaller stomata may accord benefits by being able to respond more rapidly to environmental cues than larger stomata (Drake *et al*., 2013), although this relationship does not appear to be consistent among major plant groups (McAdam & Brodribb, 2012).

One important factor driving the size of stomata is genome-related. Genome size is linked to cell sizes in both animals and plants (Cavalier-Smith, 2005; Gregory, 2005). In particular, the sizes of the guard cells of stomata and other leaf cells are correlated with genome size among angiosperms (Beaulieu *et al*., 2008). The relationship with guard cell size is of particular interest because several authors have attempted to use the size of fossil guard cells to reconstruct evolutionary trends in genome size (Franks *et al*., 2012; Lomax *et al*., 2014) or degrees of ploidy (Masterson, 1994).

The sizes of stomata and other cells in leaves are related to aspects of environment other than CO_2_. A strong association between large leaf cells and occupation of open vegetation may be related to the link between thick leaves and environments capable of supporting closed forest (Brodribb *et al*., 2013; Jordan *et al*., 2013). Furthermore, in a substantial survey of northern temperate species, very large stomata were largely restricted to herbaceous species of open vegetation (Hodgson *et al*., 2010), which is contrary to previous proposals that small stomata should be expected in open environments (Hetherington & Woodward, 2003). Furthermore, cell sizes in developmentally independent leaf tissues show a network of significant correlations that persists across a wide phylogenetic range of angiosperms, as well as life form and habitat (Brodribb *et al*., 2013; John *et al*., 2013). This suggests that there are important factors (including genome size, but also other possible factors) controlling cell size across different tissues, and that the effects of these factors have been maintained to a significant degree over many tens of millions of years. Brodribb *et al*. (2013) argued that adaptive advantages of coordinated changes in cell size in functionally linked tissues, especially the vascular system and stomata, contributed to the maintenance of these links. Thus, it appears that there are environmental drivers for coordinated variation in cell size and that changes in genome size can facilitate generic changes in cell size. There is clear evidence that genome size has both adaptive and apparently nonadaptive components (Kang *et al*., 2014).

The links between stomatal size and both genome size and environment raise the question: do plants use genome size as a mechanism for adjusting stomatal size? Although genome size change provides an apparently simple mechanism for evolutionary modification of cell size, it has a potential fitness cost because genome size appears to have a generic impact on cell size across a wide range of tissues. Although Brodribb *et al*. (2013) argued that generic changes in cell size can allow coordinated development of some disparate tissues (especially stomata and veins), it seems unlikely that the optimal cell sizes for all tissues would follow the same allometric patterns. Thus, the links between vascular tissue and stomata vary according to environmental conditions (Carins Murphy *et al*., 2013). In other words, generic changes in cell size that result in improved performance of cells in some tissues could lead to suboptimal cell sizes in other tissues. Such an imbalance could potentially lead to suboptimal overall plant function and a reduction in plant performance and fitness. This leads to a second question: are generic changes in cell size associated with changes in genome size followed by evolutionary adjustment of cell sizes in different tissues to re-establish optimal whole-plant development?

We therefore investigate the link between genome size and the size of stomata to test if there is evolutionary adjustment of genome size-related changes in cell size. We do this by comparing ancient and more recent relationships between stomatal size and genome size in the family of woody plants, Proteaceae. Because of their functional, anatomical and ecological diversity, strong fossil record and well-studied phylogeny, Proteaceae are increasingly used as a model system for understanding the evolution of plants, especially leaves (Jordan *et al*., 2005, 2008; Mast *et al*., 2008; Sauquet *et al*., 2009; Brodribb *et al*., 2013). The range in stomatal size in Proteaceae encompasses most of the range observed across land plants (Hetherington & Woodward, 2003): the guard cell length (the most widely used measure of stomatal size) in the family ranges from relatively small (mean length < 20 μm) to some of the largest known (mean length > 90 μm) (Carpenter *et al*., 2010). Furthermore, cytological studies suggest that, even though eupolyploidy is rare in the family, genome size varies markedly among genera (Stace *et al*., 1998). Finally, a significant correlation between total chromosome length and guard cell length in a small, but phylogenetically broad, sample suggests that the family conforms to the general pattern of genome size being linked to stomatal size (Brodribb *et al*., 2013). Specifically we tested whether the incorporation of habitat information (vegetation type and climate) improved predictions of stomatal size from genome size, and whether changes in stomatal size occurred after changes in genome size.

## Materials and Methods

### Species selection

The Proteaceae is an ancient family of woody, evergreen plants with over 1700 species in *c*. 80 genera (Weston, 2007). The family spans the southern hemisphere and Southeast Asia, but is centred in Australia. Proteaceae has remarkable variation in leaf form (Johnson & Briggs, 1975) matched by great diversity in leaf anatomy among species (Jordan *et al*., 2005, 2008). We sampled a total of 67 species from 48 genera (Supporting Information, Table S1). These species were selected to represent the phylogenetic, ecological, morphological and anatomical range of the family. The species included alpine, Mediterranean climate, wet heath, tropical rainforest and temperate rainforest species, and ranged in mean leaf area from < 1 cm^2^ to > 600 cm^2^. The sampling of 13 of the genera included two or more species. The number of genera with samples from multiple species was primarily constrained in two ways – many of the genera in the family only contain one species, and many of the other genera occur in areas (e.g. New Caledonia, South Africa, South America, Southeast Asia) from which it is difficult to obtain fresh material and then import it into Australia.

The plants sampled for genome size were collected from a mixture of wild-grown and cultivated plants grown from native stock. Samples were collected from a wide range of regions within Australia, ranging from tropical North Queensland to the Mediterranean climate region of Western Australia to alpine Tasmania.

### Anatomical data

The length of guard cells was measured from photomicrographs of cuticles prepared by soaking leaf samples in warm 10% aqueous Cr_2_O_3_ until clear, rinsing thoroughly, cleaning with a single-hair paintbrush if necessary, staining with dilute (*c*. 0.1%) aqueous toluidine blue, rinsing, then mounting on microscope slides in phenol glycerin jelly. Photomicrographs were taken with a Nikon Digital Sight DS-L1 camera (Melville, NY, USA) mounted on a Leica DM 1000 microscope (Nussloch, Germany). Guard cell length was measured from at least 20 cells from multiple areas within the leaf using the image analysis software, ImageJ (http://rsbweb.nih.gov/ij/index.html). Comparison of the area of leaf sample with the area of the prepared cuticle indicated that the preparation method did not result in substantial shrinkage or stretching of the cuticle.

### Environmental data

Vegetation type (open vegetation, such as heath and open woodlands vs closed forest including rainforest) followed the classification in Jordan *et al*. (2014). Although a few Proteaceae species occur in both open and closed vegetation, the allocation was more-or-less unambiguous for our sample species. Climatic distributions of species were estimated from point distribution data for 1653 species, using the WORLDCLIM 30 grid of climates (Hijmans *et al*., 2005). Point distribution records were dredged from the Global Biodiversity Information Facility (http://www.gbif.org/), the Atlas of Living Australia (http://ala.org.au/) and supplemented by personal observations where possible.

### Genome sizes

Genome sizes were estimated from samples (*c*. 1 cm^2^) of expanding foliage. Incompletely expanded leaves were used because of the highly sclerophyllous nature of many Proteaceae. For each sample, runs were replicated on different days. The samples of the target species and reference plant were co-chopped with a double-sided razor blade in a precooled Petri dish containing 200 μl woody plant buffer (WPB, Loureiro *et al*., 2007b) with 3% PVP-10 (WPB2) as per Beatson *et al*. (2003). From these preparations, nuclei were isolated and stained using the Cystain® PI Absolute P Kit (Partec, Munster, Germany) with the following modification: the kit extraction buffer was replaced with WPB2. An additional 200 μl of WPB2 was added and the nuclei suspension filtered through a 20 μm Celltrics® filter (Partec). Before analysis, the suspension was incubated for between 30 and 60 min, with 1.6 ml staining buffer containing propidium iodide (PI) and RNAse. The genome size of each species was determined on a CyFlow Ploidy Analyser (Partec) with DNA-PI script parameters: L-L 0.30, speed 0.4 μl s^−1^ and gain *c*. 360.

*Pisum sativum* ‘Torstag’ (2*x* = 9.49 pg) was used as the reference plant for all samples except those of *Bellendena montana*. The peaks for *B. montana* and *P. sativum* were very close, so using *P. sativum* as reference would lead to ambiguity in the estimated genome size. A single cultivated plant of *Acacia auriculiformis* (2*x* = 1.77 pg) was used as a reference for *B. montana*. At least two estimates were made for each species on different days.

The nuclear size was estimated according to the formula of Loureiro *et al*. (2007a): 

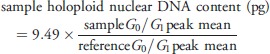
Eqn 1

To ensure consistency with the Kew Database of genome sizes, reference values were determined based on 10 runs relative to a diploid nuclear value of 9.56 pg for *P. sativum* ‘Minerva Maple’ (Galbraith & Lambert, 2012).

### Phylogeny and analyses

We generated four data sets that allowed independent analysis of patterns at higher and lower taxonomic levels, and also allowed for the potential effects resulting from the presence of the different genome size–guard cell size relationship observed in the Persoonioid clade (subfamilies Persoonioideae and Bellendenoideae) and Grevilleoid/Proteoid clade (i.e. the whole family excluding the Persoonioid clade). Thus, we analysed high-level relationships (among genera or high-level clades within two large genera) for the whole family and for the Grevilleoid/Proteoid clade. These two data sets therefore reflected the effects of ancient evolutionary processes. The second group of data sets contained data from multiple species within genera or high-level clades within genera, and were analysed to extract information on more recent processes. Again, we analysed both data from across the whole family, and data from just the Grevilleoid/Proteoid clade. Genome size, precipitation of the driest quarter and guard cell length data all showed skewed distributions and were log-transformed in all four data sets before analysis to ensure that the assumptions of the analyses were valid. For guard cell size, we had two replicate plants for each species – one being the individual from which genome size was measured and the other a plant from a different environment (e.g. where the plant measured for genome size was cultivated, we used a field-grown plant as the replicate).

**Figure 1:**
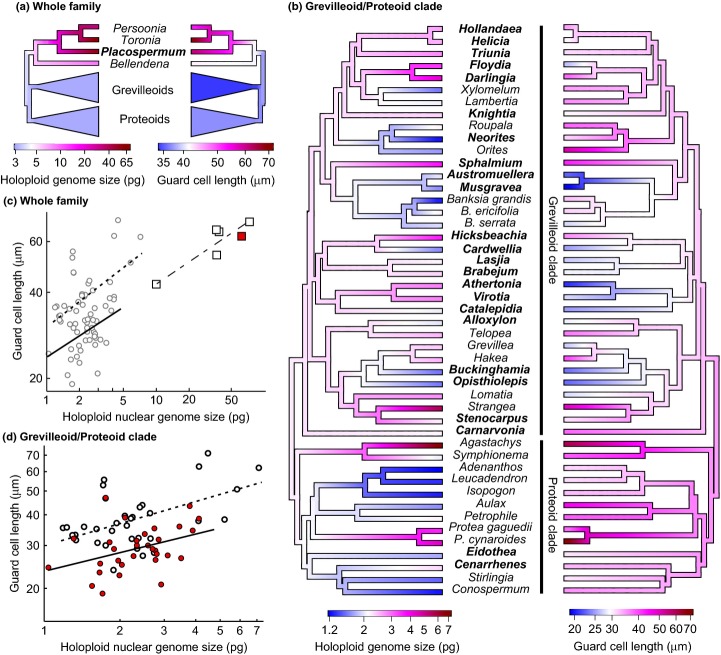
Holoploid genome size and guard cell length in Proteaceae (all plots are on log scales). (a, b) Maximum likelihood ancestral state reconstructions of holoploid genome size and guard cell length performed using the contMap command of phytools in R (Revell, 2014). Branch lengths are proportional to molecular dates (Sauquet *et al*., 2009). Species from genera in bold are from closed forest. (a) Whole family, with Grevilleoid and Proteoid clades collapsed. (b) Grevilleoid/Proteoid clade. (c, d) The relationship of guard cell length to genome size and vegetation type. (c) Whole family, highlighting the Persoonioid clade (open squares, open vegetation; closed squares, closed forest) with a power regression for open vegetation in that subfamily (rest of the family shown in circles, with regression lines from (d)). Note that the open vegetation Persoonioids have much larger genomes relative to guard cell length than open vegetation species in the rest of the family. (d) The Grevilleoid/Proteoid clade, with separate power regressions for open vegetation (open circles) and closed forest (closed circles). Note that open vegetation species have longer guard cells relative to genome size than closed forest species.

For the high-level analyses, we generated a phylogeny (Fig.1) by reducing the dated phylogeny of Sauquet *et al*. (2009) to terminals for which we had genome size data, and adding one terminal, *Lasjia*. The genus *Lasjia* was not included in the phylogeny of Sauquet *et al*. (2009) but was present in a dated phylogeny of tribe Macadamiaeae (Mast *et al*., 2008). The phylogenies of Sauquet *et al*. (2009) and Mast *et al*. (2008) provided similar age estimates for the shared node *Macadamia* + *Brabejum*, the node that is proximal to *Lasjia* + *Macadamia* in Mast *et al*. (2008). We therefore estimated the age of *Lasjia* by interpolating between the age of the *Macadamia* + *Brabejum* node and the terminal nodes. The whole-family data had 51 species from 48 genera, and the Grevilleoid/Proteoid data had 47 species from 44 genera. Of the 51 species we measured, 34 were the same as species used in the phylogeny of Sauquet *et al*. (2009). For another four of our species, there is phylogenetic evidence that they are closely related to the species in the phylogeny (Mast & Givnish, 2002; Valente *et al*., 2010; Mast *et al*., 2012), and the remaining 12 species were assigned by assuming the monophyly of genera.

We used a multiple regression-type approach to test for adaptive adjustment of the genome size–cell size relationship in both high-level data sets. We used phylogenetic generalised least-squares regressions implemented using the pgls command of the package caper (Orme *et al*., 2013) in R, which allows both continuous and categorical predictors to be modelled. Analyses were performed with unconstrained values of *λ*. We predicted log of guard cell length using all combinations of vegetation type, log of genome size and log of dry season precipitation, and selected the best models using Aikake's information criterion (AIC). We used *F*-tests from the models to test whether individual predictors contributed significantly to the prediction of log of guard cell length. Similar analyses using standard least-squares analyses (i.e. not phylogenetically adjusted) produced closely comparable results to the phylogenetically adjusted methods and will not be presented here. We analysed both the mean guard cell length of the two replicates of each species and the mean guard cell length of the individual measured for genome size. These approaches differ in that the analyses based on the means of replicate samples within species assume that genome sizes are constant within species, but provide the advantage of removing the effects of plasticity within species. The analyses based on a single sample come at the cost of not allowing for plastic variation in stomatal size. The single-sample analysis yielded qualitatively the same results as the analyses-based means of replicates within species, but resulted in poorer statistical fits, suggesting that the potential error induced by within-species differences in genome size was outweighed by the reduction of error resulting from plastic responses to environment in guard cell length. We therefore focus on analyses based on the means of the two replicate samples.

The low taxonomic-level data contained replicate species from genera, with each genus being represented by one species from the high-level data and one or two other species. The whole family data set had 13 genera (28 species in total), whereas the Grevilleoid/Proteoid clade data set had 12 genera and 25 species. These low taxonomic level data sets were analysed with variance components analyses based on a maximum likelihood analysis of a nested design of random effects of genus, species-within-genera, and replicates-within-species. These analyses were implemented in LME4 (Douglas Bates *et al*., 2014) and HLMdiag (Loy, 2014) in R. SE of variance components were estimated using the Mixed procedure of SAS 9.2 (SAS Institute Inc., Cary, NC, USA). For genome size, the replicates were the duplicate runs from the same plant. For guard cell size, the replicates were from different plants from the same species from different environments. Variance components analysis of nested samples identifies the variance uniquely attributable to each level of the hierarchy. In other words, the among-genus effect reflects the variation among genera independent of the variation within genera, and the among-species-within-genus effect reflects the variation at this level independent of the variation among genera or within species. Data from one known neopolyploid, the triploid *Lomatia tasmanica* (Lynch *et al*., 1998), were excluded from these data sets because of the rarity of polyploidy in the family, and because the work focuses on events in deeper time.

## Results

### Overall patterns in genome size and guard cell size

The estimates of genome size were highly repeatable, with the pooled SD of the log_10_ of holoploid genome size from the replicate analyses within species being 0.0194. This is equivalent to an SD of *c*. 4.5% of the observed genome size. Inferred monoploid (1C) genome size varied widely, ranging from 0.52 to 30.14 pg (Table S1), which is consistent with available cytological evidence of chromosome lengths (Stace *et al*., 1998). However, all but six species had small genomes, with 1C ranging from 0.5 to 5 pg (Table S1). Stomata also varied widely in size, with mean guard cell length ranging from 19.1 to 71.5 μm.

**Figure 2:**
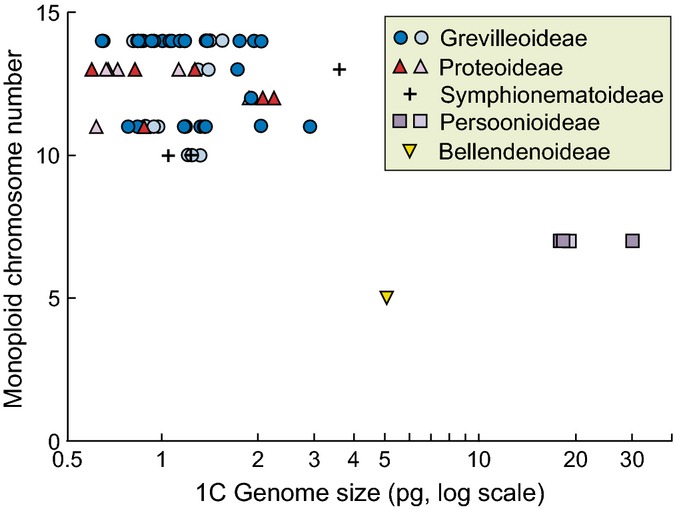
Monoploid genome size (1C; plotted on a log scale) vs monoploid chromosome number (1x) for Proteaceae species. Note the large variation in genome size and the lack of positive relationship with chromosome number. Chromosome numbers are based on counts documented in Stace *et al*. (1998), with counts for either the same species (dark symbols) or other members of the relevant genus (pale symbols; see also Table S1). Thus, variation in genome size in Proteaceae is largely driven by chromosome size.

There was no relationship between haploid chromosome number and monoploid genome size (Table S1; Fig.2). However, the two known eupolyploids for which data were available to us – triploid *L. tasmanica* (Lynch *et al*., 1998) and tetraploid *Toronia toru* (Stace *et al*., 1998) – showed the expected patterns in genome size. The 1C values of these species were 1.37 and 17.87 pg, respectively, which were very similar to those of their respective diploid relatives (other *Lomatia* species, 1.35–1.38 pg; *Persoonia* species, 17.62–18.67 pg).

### Phylogenetic patterns in genome size and guard cell size

Both holoploid genome size and guard cell length showed significant phylogenetic signals (Table1). For genome size, this signal was very strong both across the whole family and within the Grevilleoid/Proteoid clade, and for guard cell length the signal was very strong across the whole family according to both metrics, and significant for the Grevilleoid/Proteoid clade when measured using Pagel's *λ* but not Blomberg's *K*.

**Table 1:** Tests for phylogenetic signal in the high taxonomic level data, representing among-genera variation

Parameter	Whole family				Grevilleoid/Proteoid clade			
	*K*	*P*-value	*λ*	*P*-value	*K*	*P*-value	*λ*	*P*-value
Log of holoploid genome size	1.93	**< 0.0001**	0.99	**< 0.0001**	0.79	**0.003**	0.99	**0.02**
Log of guard cell length	0.90	**0.002**	0.71	**< 0.001**	0.65	0.08	0.53	**0.04**

Blomberg's *K* and Pagel's *λ* were calculated using the phylosig command in phytools in R (Revell, 2014). Statistically significant values are indicated in bold text.

Holoploid genome size varied considerably across the phylogeny (Fig.1a,b), with conspicuously large genomes in the Persoonioid clade (subfamilies Persoonioideae and Bellendenoideae) (2C > 10 pg), but also relatively large genomes in *Agastachys* and *Strangea*. Guard cell size also varied across the phylogeny, with very large stomata in Persoonioideae and in several disparate clades within the Grevilleoid/Proteoid clade – *Protea*,*Agastachys*,*Orites*,*Strangea* and *Sphalmium* (Fig.1a,b). Ancestral state analysis suggests that the family's ancestral genome size was intermediate (2C of *c*. 5 pg), and increased in the Persoonioid clade, with most changes in the Grevilleoid/Proteoid clade being towards smaller genomes (Fig.1). Similarly, ancestral state analysis suggests that the ancestral guard cell length was relatively large (*c*. 45 μm), with increases in the Persoonioid clade and both increases and decreases in the Grevilleoid/Proteoid clade. However, these inferences should be treated with caution because of potential biases as a result of directional evolution (Oakley & Cunningham, 2000). This is particularly possible in Proteaceae, given the presence of a major trend from closed forest in the Eocene to open vegetation more recently (Kershaw *et al*., 1994). By contrast, phylogenetically adjusted regressions, such as those used in this study, are more robust to biases resulting from directional evolution, presumably because the biases operate similarly on both dependent and independent variables (Oakley & Cunningham, 2000).

Although there were highly significant positive correlations between log of guard cell length and log of holoploid genome size (phylogenetically adjusted *R *=* *0.61 for the whole family; 0.53 for the Grevilleoid/Proteoid clade; *P *<* *0.001 in both cases) these relationships were relatively weak. Importantly, the best phylogenetically corrected models for the guard cell length–holoploid genome size relationship also included highly significant contributions of vegetation type both across the whole family and within the Grevilleoid/Proteoid clade (*P *<* *0.001; Table2). Thus for each of these two data sets, the two best models included the effects of both holoploid genome size and vegetation type, and differed only in the presence or absence of precipitation of the driest quarter as a predictor. In particular, closed forest had considerably smaller guard cells than open vegetation for a given holoploid genome size (Fig.1d). Although precipitation of the driest quarter was included in the best model in the Grevilleoid/Proteoid clade, this effect was not significant (*P *>* *0.05), arguing that the case for climate influencing guard cell length is ambiguous. The two best models gave much better fits (AIC values >* *10 lower) than the model predicting guard cell size from holoploid genome size alone. Relative to stomatal size, the genomes of open vegetation species were much larger in the Persoonioid clade than in the Grevilleoid/Proteoid clade (Fig.1c). Valid comparisons could not be made for closed forest species because Persoonioids have only one true closed forest species (*Placospermum coriaceum*).

**Table 2:** The three best phylogenetically corrected models predicting guard cell length from genome size and climate

Model				AIC
Whole family				
1	Genome size ***	Vegetation type ***		−112.43
2	Genome size ***	Vegetation type ***	Dry season precipitation (NS)	−111.66
3	Genome size ***	Vegetation type ***	Vegetation type|genome size (NS)	−110.56
Grevilleoid/Proteoid clade				
1	Genome size ***	Vegetation type ***	Dry season precipitation (NS)	−105.03
2	Genome size ***	Vegetation type ***		−105.02
3	Genome size ***	Vegetation type ***	Vegetation type|genome size (NS)	−104.18

In each model, guard cell length, genome size and dry season precipitation were all log-transformed. All other models had Akaike's information criterion (AIC) values at least 2 greater than the best model, indicating that these models provided much poorer fits than the best model. Probabilities for each effect are indicated (NS (not significant), *P *>* *0.05; ***,*P *<* *0.001).

### Patterns within genera

**Figure 3:**
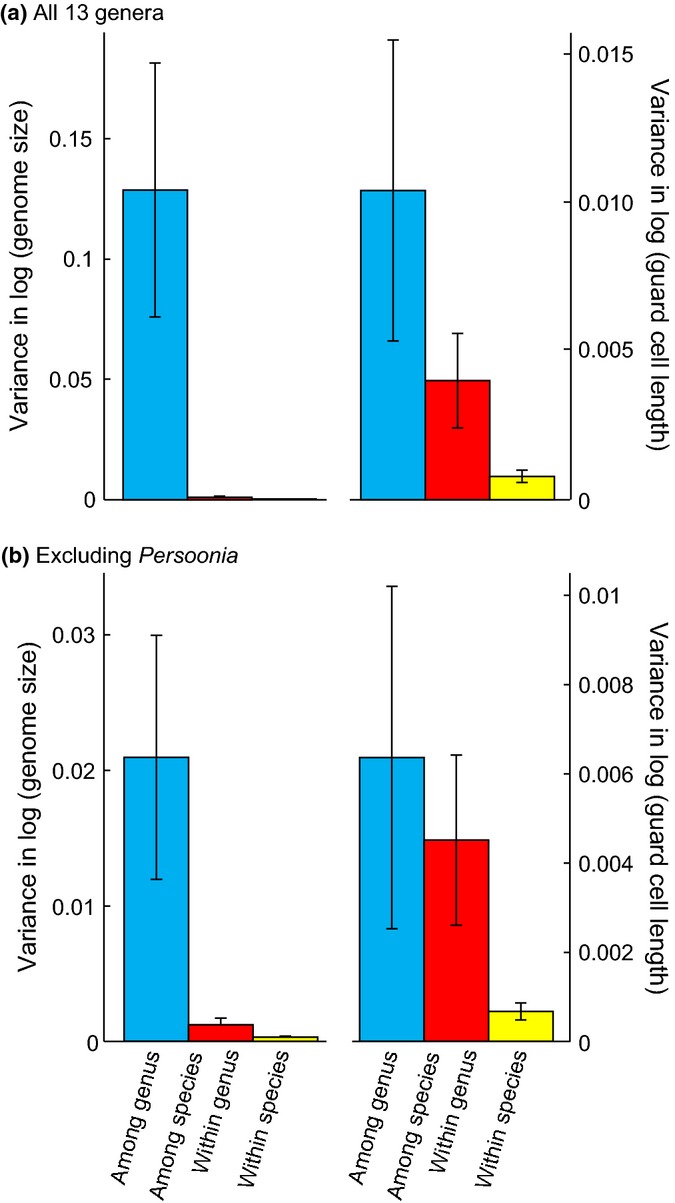
Variation in log of holoploid genome size and log of guard cell length partitioned among genera, among species within genera and within species. Values are the components of variance uniquely attributable to each level in the hierarchy. (a) Thirteen genera from all subfamilies; (b) 12 genera from the Grevilleoid/Proteoid clade. Error bars, SE.

Genome size varied very little within genera, with almost 99% of the variation in 1C size occurring among genera and < 1% occurring among species within genera (Fig.3). When the Persoonioid clade, with its anomalously large genome, was excluded, 93.2% of the variation still occurred among genera, and only 5.5% among species within genera. In contrast to genome size, stomatal size showed high amounts of variation within genera relative to the variation among genera (Fig.3). Thus, 69% of the variation in log of guard cell length occurred among species within genera, 26% occurred among genera and 5% occurred between individuals within species. When the Persoonioid clade was excluded, 55% of the variation occurred among species within genera, 39% among genera and 6% between individuals within species (Fig.3). As a proportion of the variance among genera, the among-species-within-genera variance component for log of guard cell length was significantly greater than for holoploid genome size, both across the whole family and within the Grevilleoid/Proteoid clade (*P *<* *0.01; randomization test based on 9999 replicates of resampling genera with substitution). As expected, the recently evolved triploid, *L. tasmanica* (excluded from analyses), had slightly larger stomata than its congeners, but the more ancient tetraploid (*T. toru*) had stomata of similar size to those of its closest relatives, *Persoonia* species, in spite of having double the genome size.

## Discussion

The analyses clearly show that stomatal size is best predicted by a combination of genome size and vegetation structure, with a possible contribution of climate. Furthermore, the high amounts of within-genus variation in guard cell size and lack of variation in genome size at this level suggest that substantial evolutionary changes in stomatal cell size occurred after most major changes in genome size. Taken together, these results provide strong evidence that stomatal size–genome size relationships are adjusted by evolutionary adaptation to the macroenvironment.

### Variation in genome size

Holoploid genome sizes observed in Proteaceae ranged from *c*. 1 to > 70 pg (*c*. 1000 to *c*. 70 000 megabase pairs). Almost all of this 70-fold range reflected evolutionary changes at relatively high taxonomic levels (among genera or among groups of genera) with < 7% of the observed variation in genome size occurring within genera (Fig.3). This implies that substantial changes in genome size are relatively rare. Because our genome size data did not include replicate individuals within species, the among-species-within-genera variance component includes information from both the among-species and among-individuals-within-species variances. The small size of the among-species-within-genera component therefore means that the true individuals-within-species component must be very small. This is consistent with the view that genome size varies little within species unless mediated by polyploidy (Greilhuber, 1998).

The most spectacular changes in genome size were related to the very large chromosomes in subfamilies Persoonioideae and Bellendenoideae, as observed by Stace *et al*. (1998). Among eudicots, the mean chromosome size of *Placospermum* (*c*. 4.3 pg) was only exceeded in the Kew database of genome sizes (http://data.kew.org/cvalues/, accessed 28 March 2014) by three species of *Viscum* (Santalaceae). The chromosomes of other Persoonioideae (mean of *c*. 2.5 pg) were also among the top 1% for eudicots, and those of Bellendenoideae were intermediate in size (*c*. 1 pg) between Persoonioideae and other Proteaceae. The chromosomes of species in the Grevilleoid/Proteoid clade, representing the rest of the family, were small to medium-sized but still varied more than fivefold (0.05–0.27 pg).

Genome size had no discernible relationship with chromosome number (Fig.2) apart from known cases of eupolyploidy (tetraploid *T. toru* and triploid *L. tasmanica*), which showed expected increases in holoploid genome size compared with their diploid relatives. Instead, the variation in holoploid genome size was almost entirely explained by variation in chromosome size (*R*^2^ = 0.996 for the 43 species with known chromosome counts and *R*^2^ = 0.948 excluding members of the Persoonioid clade). Although it is possible that this variation in chromosome size is the result of complex processes of past polyploidization followed by chromosome fusion, this seems unlikely given that chromosome sizes vary considerably within clades with consistent chromosome numbers (Fig.2). Thus, mechanisms other than ploidy change (Leitch & Leitch, 2012) appear to be the main drivers of variation in chromosome size in Proteaceae.

### Evolutionary adjustment of the relationships of cell sizes to genome sizes

The significant phylogenetically independent association between genome size and guard cell length in Proteaceae is entirely consistent with broad patterns across land plants (Beaulieu *et al*., 2008). However, close inspection of the relationship clearly shows that much of the genome-size related variation in stomatal size is modified by subsequent adaptation. The strong environmental signals in the size of stomata independent of the effects of genome size (Fig.1c,d; Table2) provides evidence that stomatal size has an adaptive component that is independent of generic impacts of changes in genome size. The large differences in stomatal size within clades with stable genome sizes imply repeated adjustment of stomatal size subsequent to the major changes in genome size (Fig.3). Although Hodgson *et al*. (2010) argue that the cell size–genome size relationship can operate in both directions (i.e. genome size driving cell size, or cell size driving genome size), our results are consistent with the former direction.

In contrast to the very high stability level of genome size within genera, stomatal size varied markedly at this level (Fig.3). These differences among species in stomatal size can be largely attributed to evolutionary changes. First, because our guard cell length data include the variation among different individuals grown under a range of conditions, the within-species guard cell length variance component captures the plastic responses among individuals within the species (as well as some genetic variation). As a result, the among-genera and among-species-within-genera components will be virtually entirely attributable to genetic variation. Furthermore, our observed range in guard cell lengths within genera (23–66 μm) is approximately an order of magnitude greater than the differences induced within genotypes of *Arabidopsis thaliana* across a wide range of physiologically significant environmental conditions (Lomax *et al*., 2009). Finally, specimens from different plants of the same species of Proteaceae in a survey of *c*. 600 species of Proteaceae (Jordan *et al*., 2014) indicate little variation in guard cell length within species.

The occurrence of very large stomata in *Orites milliganii* and *Orites acicularis* represent a clear example of modification of stomatal size independent of genome size. These species have stomata that are *c*. 40–100% longer than other members of their tribe (Roupaleae) but have smaller genomes than 12 of the 13 other sampled members of Roupaleae (Fig.1b). Similarly, both very large and small stomata occur in *Protea*, with only small changes in genome size (Fig.1b). In addition, two major transitions in genome size within Persoonioideae were not associated with substantial changes in stomatal size. Both *P. coriaceum* and *T. toru* have much larger genomes than other Persoonioideae, but the guard cells of all measured Persoonioideae are of similar size. Thus, the guard cells of *Persoonia* (> 50 μm long) are among the larger stomata of land plants, but increases in stomatal size in *P. coriaceum* and *T. toru* in proportion to their genome sizes (relative to other Persoonioideae) would result in giant guard cells that may exceed functional limits in guard cell size.

The regression modelling clearly indicated that incorporating habitat characteristics (especially vegetation type) provided considerably better predictions of both guard cell length than models based solely on genome size. This implies that, in Proteaceae at least, large changes in stomatal size can and do evolve in response to the environment without changes in genome size. The adjustment of stomatal size in response to vegetation type (Fig.1d) implies that some aspect of the difference between open vegetation and closed vegetation is linked to selection for stomatal size. This may be a direct response to vegetation structure, with the obvious candidate being low light intensities in the understorey of closed forest. High radiation intensities are strongly associated with thick leaves (Mott *et al*., 1982) and Brodribb *et al*. (2013) argued that increased cell size is a simple mechanism for creating a thick leaf. In addition, open vegetation species of Proteaceae generally occur in environments that are too nutrient-poor, too cold in summer or too dry to support closed forest – that is, conditions favouring the ‘slow’ end of the leaf economics spectrum (Wright *et al*., 2004). As a result, leaf economics may contribute to the presence of large stomata in open vegetation, particularly given that having large stomata may come at the cost of reduced maximum photosynthetic capacity (Franks & Beerling, 2009). This reduction occurs because stomatal size is inversely related to stomatal density, a relationship that is particularly strong in Proteaceae (Brodribb *et al*., 2013). The impacts of direct responses to radiation intensities and indirect responses via leaf economics are not mutually exclusive. In particular, the benefits of having thick leaves may contribute to offsetting the costs in productivity associated with large stomata.

### Implications for using fossil stomata as proxies for past genome size and atmospheric CO_2_

The habitat type and clade-related variation in the guard cell-size–genome size relationship and the large deviations from the regressions within clades and habitat types impact on the use of fossil guard cell size in predicting past genome sizes. The central problem is that vegetation type and possibly other factors such as atmospheric CO_2_ concentration affect stomatal size independently of genome size. Thus, a general guard cell size–genome size relationship cannot be expected through geological time because of changes in vegetation type (Scott, 2000) and atmospheric CO_2_ concentrations (Berner & Kothavala, 2001) over the last 90 million yr, and more. This kind of problem represents a strong and often overlooked cause of error in using fossils as proxies for past environments (Jordan, 2011). For example, the use of *Ginkgo* stomata as a proxy for palaeo-CO_2_ assumes that the stomata–environment relationship underpinning the proxy has not been affected by extinction or evolution, even though the calibration group (extant *Ginkgo biloba*) is only a tiny subset of the past diversity of the species or genus (Jordan, 2011). In this context, the results presented here should not be ignored as a special case because of the very broad ranges in both guard cell size and genome size in Proteaceae. More accurate predictions of genome size from fossil stomata may be achieved by adjusting the guard cell size–genome size relationship to allow for vegetation type, as inferred from proxy evidence (Jordan *et al*., 2014). Close investigation of the environment–guard cell size–genome size relationships may allow further improvements in these predictions. Although older methods for predicting palaeo-CO_2_ from stomata employed the abundance of stomata (either as stomatal index or, less frequently, as stomatal density) and may be biased by the effects of changes in stomatal size on gas exchange, more recent models incorporate stomatal size (Grein *et al*., 2013; Franks *et al*., 2014). The presence of strong environmental adaptation in stomatal size leads to optimism with regard to the more advanced uses of fossil stomata as proxies for past atmospheric CO_2_.

## Supporting Information

**Table S1** Species analysed, including mean holoploid genome size
