# Supplementary material for: Environmental adaptation in stomatal size independent of the effects of genome size
Source: New Phytol. 2014 Sep 30;205(2):608–17. doi: 10.1111/nph.13076 (PMC4301182; doi:10.1111/nph.13076)
Supplement: Supplementary file 1 — Table S1 Species analysed, including mean holoploid genome size [file nph0205-0608-sd1.docx]

**Supporting Information Table S1** Species analysed, including mean holoploid genome size (± SE, estimated from the pooled variance of the log of genome size)

| **Subfamily** | **Species** | | **Holoploid genome size (pg ± SE)** | **1C (pg)** | **Habitat** | **Chromosome number - ploidy** |
| --- | --- | --- | --- | --- | --- | --- |
| **Species in phylogenetic datasets (species with asterisks were used in the low taxonomic level analyses)** | | | | | |  |
| Grevilleoideae | | **Alloxylon flammeum* P.H. Weston & Crisp | 1.79 ± 0.06 | (0.90) | tropical rainforest | (22 - 2n) |
| Grevilleoideae | | *Athertonia diversifolia* L.A.S. Johnson & B. G. Briggs | 2.85 ± 0.09 | (1.43) | tropical rainforest | (28 - 2n) |
| Grevilleoideae | | *Austromuellera trinervia* C.T. White | 1.82 ± 0.05 | 0.91 | tropical rainforest | 28 - 2n |
| Grevilleoideae | | **Banksia ericifolia* L.f. | 1.27 ± 0.04 | 0.64 | dry sclerophyll woodland | 28 - 2n |
| Grevilleoideae | | **Banksia grandis* Willd. | 2.19 ± 0.07 | 1.10 | dry sclerophyll woodland | 28 - 2n |
| Grevilleoideae | | **Banksia serrata* L.f. | 1.83 ± 0.06 | 0.91 | dry sclerophyll woodland | 28 - 2n |
| Grevilleoideae | | *Brabejum stellatifolium* L. | 2.30 ± 0.08 | 1.15 | temperate forest | 28 - 2n |
| Grevilleoideae | | *Buckinghamia celsissima* F. Muell. | 1.63 ± 0.05 | 0.82 | tropical rainforest | 22 - 2n |
| Grevilleoideae | | *Cardwellia sublimis* F. Muell. | 1.64 ± 0.05 | 0.82 | tropical rainforest | 28 - 2n |
| Grevilleoideae | | *Carnarvonia araliifolia* F. Muell. | 2.05 ± 0.07 | 1.03 | tropical rainforest | 28 - 2n |
| Grevilleoideae | | *Catalepidia heyana* (F.M. Bailey) P.H. Weston | 2.00 ± 0.07 | (1.00) | tropical rainforest | (28 - 2n) |
| Grevilleoideae | | **Darlingia darlingiana* (F. Muell.) L.A.S. Johnson | 3.82 ± 0.12 | 1.91 | tropical rainforest | 28 - 2n |
| Grevilleoideae | | *Floydia praealta* (F. Muell.) L.A.S. Johnson & B. G. Briggs | 3.44 ± 0.11 | 1.72 | subtropical rainforest | 28 - 2n |
| Grevilleoideae | | *Grevillea australis* R. Br. | 2.42 ± 0.08 | (1.21) | montane heath | (20 - 2n) |
| Grevilleoideae | | **Hakea salicifolia* (Vent.) B.L. Burtt | 2.59 ± 0.08 | (1.29) | temperate forest | (20 - 2n) |
| Grevilleoideae | | *Helicia australasica* F. Muell. | 2.54 ± 0.08 | 1.27 | tropical rainforest | 28 - 2n |
| Grevilleoideae | | *Hicksbeachia pinnatifolia* F. Muell. | 3.39 ± 0.11 | 1.69 | tropical rainforest | 26 - 2n |
| Grevilleoideae | | **Hollandaea riparia* B. Hyland | 2.69 ± 0.09 | (1.34) | tropical rainforest | (28 - 2n) |
| Grevilleoideae | | *Knightia excelsa* R. Br. | 2.29 ± 0.08 | 1.15 | temperate forest | 28 - 2n |
| Grevilleoideae | | *Lambertia formosa* Sm. | 2.03 ± 0.07 | 1.05 | dry sclerophyll woodland | 28 - 2n |
| Grevilleoideae | | **Lomatia tinctoria* (Labill.) R.Br. | 2.43 ± 0.08 | 1.22 | dry sclerophyll woodland | 22 - 2n |
| Grevilleoideae | | *Lasjia grandis* | 2.69 ± 0.09 | (1.34) | subtropical rainforest | (28 - 2n) |
| Grevilleoideae | | *Musgravea heterophylla* L.S. Smith | 1.68 ± 0.05 | 0.84 | tropical rainforest | 28 - 2n |
| Grevilleoideae | | *Neorites kevedianus* L.S. Smith | 1.29 ± 0.04 | 0.64 | tropical rainforest | 28 - 2n |
| Grevilleoideae | | *Opisthiolepis heterophylla* L.S. Smith | 1.52 ± 0.05 | 0.76 | tropical rainforest | 22 - 2n |
| Grevilleoideae | | **Orites acicularis* (R. Br.) Roem. & Shult. | 1.68 ± 0.06 | 0.84 | montane heath | 28 - 2n |
| Grevilleoideae | | *Roupala pseudocordata* Pittier | 2.17 ± 0.07 | (1.09) | montane heath | (28 - 2)n |
| Grevilleoideae | | *Sphalmium racemosum* (C.T. White) B.G. Briggs, B. Hyland & L.A.S. Johnson | 3.72 ± 0.12 | 1.86 | tropical rainforest | 24 - 2n |
| Grevilleoideae | | **Stenocarpus sinuatus* (Loudon) Endl. | 2.33 ± 0.07 | 1.17 | tropical rainforest | 22 - 2n |
| Grevilleoideae | | *Strangea linearis* Meisn. | 5.73 ± 0.19 | 2.87 | dry sclerophyll woodland | 22 - 2n |
| Grevilleoideae | | *Telopea truncata* (Labill.) R.Br. | 2.32 ± 0.08 | 1.16 | temperate forest | 22 - 2n |
| Grevilleoideae | | *Triunia youngiana* (C.T. Moore&F. Muell. ex F. Muell.)L.A.S. Johnson&B. G. Briggs | 2.80 ± 0.09 | (1.40) | subtropical rainforest | (28 - 2n) |
| Grevilleoideae | | *Virotia leptophylla* (Guillaum.) L.A.S. Johnson & B.G.Briggs | 2.75 ± 0.09 | (1.38) | tropical rainforest | (26 -2n) |
| Grevilleoideae | | *Xylomelum salicinum* (Meisn.) Benth. | 1.59 ± 0.05 | (0.79) | dry sclerophyll woodland | (28 - 2n) |
| Proteoideae | | *Adenanthos sericeus* Labill. | 1.17 ± 0.04 | 0.59 | dry sclerophyll woodland | 26 - 2n |
| Proteoideae | | *Aulax cancellata* (L.) Druce | 1.72 ± 0.06 | 0.86 | dry sclerophyll woodland | 22 - 2n |
| Proteoideae | | *Cenarrhenes nitida* Labill. | 2.46 ± 0.08 | 1.23 | temperate forest | 26 - 2n |
| Proteoideae | | *Conospermum taxifolium* C.F. Gaertn. | 1.21 ± 0.04 | (0.61) | dry sclerophyll woodland | (22 - 2n) |
| Proteoideae | | *Eidothea hardeniana* A.W. Douglas & B. Hyland | 1.81 ± 0.05 | (0.91) | subtropical rainforest | (2n) |
| Proteoideae | | **Isopogon ceratophyllus* R. Br. | 1.30 ± 0.04 | (0.65) | dry sclerophyll woodland | (26 - 2n) |
| Proteoideae | | *Leucadendron salignum* P.J. Bergius | 1.31 ± 0.04 | (0.66) | dry sclerophyll woodland | (26 - 2n) |
| Proteoideae | | *Petrophile shirleyae* F. M. Bailey | 2.20 ± 0.07 | (1.10) | dry sclerophyll woodland | (26 - 2n) |
| Proteoideae | | *Protea cynaroides* (L.) L. | 4.40 ± 0.14 | 2.20 | dry sclerophyll woodland | 24 - 2n |
| Proteoideae | | **Protea gaguedii* J.F. Gmel. | 3.69 ± 0.12 | (1.85) | dry sclerophyll woodland | (24 - 2n) |
| Proteoideae | | *Stirlingia latifolia* (R. Br.) Steud. | 1.60 ± 0.05 | 0.80 | dry sclerophyll woodland | 26 - 2n |
| Symphionematoideae | | *Agastachys odorata* R. Br. | 7.04 ± 0.22 | 3.52 | wet heath | 26 - 2n |
| Symphionematoideae | | **Symphionema montanum* R. Br. | 2.06 ± 0.07 | 1.03 | dry sclerophyll woodland | 20 - 2n |
| Bellendenoideae | | *Bellendena montana* R. Br. | 9.91 ± 0.31 | 4.95 | montane heath | 10 - 2n |
| Persoonioideae | | **Persoonia linearis* R. Br. | 35.02 ± 0.93 | 17.51 | dry sclerophyll woodland | 14 - 2n |
| Persoonioideae | | *Placospermum coriaceum* C.T. White & W.D. Francis | 60.62 ± 1.90 | 30.31 | tropical rainforest | 14 - 2n |
| Persoonioideae | | *Toronia toru* (A. Cunn.) Johnston & Briggs | 70.22 ± 2.29 | 35.11 | temperate forest | 28 - 4n |
| **Additional species used for within-genus analyses** | | | | | |  |
| Grevilleoideae | | *Alloxylon pinnatum* (Maiden & Betche) P.H. Weston & Crisp | 1.72 ± 0.06 | (0.86) | subtropical rainforest | (22 - 2n) |
| Grevilleoideae | | *Banksia marginata* Cav. | 1.72 ± 0.06 | (0.86) | dry sclerophyll woodland | (28 - 2n) |
| Grevilleoideae | | *Darlingia ferruginea* J.F. Bailey | 4.04 ± 0.13 | 2.02 | tropical rainforest | 28 - 2n |
| Grevilleoideae | | *Hakea lissosperma* R. Br. | 2.36 ± 0.08 | (1.18) | temperate forest | (20 - 2n) |
| Grevilleoideae | | *Helicia nortoniana* (F.M. Bailey) F.M. Bailey | 2.72 ± 0.09 | 1.36 | tropical rainforest | 28 - 2n |
| Grevilleoideae | | *Hollandaea sayeriana* (F. Muell.) L.S. Smith | 2.71 ± 0.09 | 1.36 | tropical rainforest | 28 - 2n |
| Grevilleoideae | | *Lomatia fraxinifolia* F. Muell. ex Benth. | 2.64 ± 0.09 | 1.32 | tropical rainforest | 22 - 2n |
| Grevilleoideae | | *Lomatia polymorpha* R. Br. | 2.60 ± 0.09 | 1.30 | temperate forest | 22 - 2n |
| Grevilleoideae | | *Orites milliganii* Meisn. | 1.70 ± 0.06 | (0.85) | montane heath | (28 - 2n) |
| Grevilleoideae | | *Stenocarpus verticis* Foreman | 1.91 ± 0.06 | (0.95) | dry sclerophyll woodland | (22 - 2n) |
| Proteoideae | | *Isopogon cuneatus* R. Br. | 1.42 ± 0.05 | (0.71) | dry sclerophyll woodland | (26 - 2n) |
| Proteoideae | | *Protea neriifolia* R. Br. | 4.05 ± 0.13 | 2.03 | dry sclerophyll woodland | 24 - 2n |
| Symphionematoideae | | *Symphionema paludosum* R. Br. | 2.41 ± 0.08 | 1.21 | dry sclerophyll woodland | 20 - 2n |
| Persoonioideae | | *Persoonia gunnii* Hook. f. | 36.64 ± 1.18 | (18.32) | montane heath | (14 - 2n) |
| Persoonioideae | | *Persoonia laurina* Pers. | 34.62 ± 1.11 | 17.31 | temperate forest | 14 - 2n |
| **Additional species scored but excluded from analyses** | | | | | |  |
| Grevilleoideae | *Lomatia tasmanica* W. M. Curtis | | 4.02 ± 0.13 | 2.01 | temperate forest | 33 - 3n |

Inferred 1C (monoploid) genome size and chromosome numbers are included, based on chromosome counts of that species ([Stace *et al.*, 1998](#_ENREF_44)) except those species with values in parentheses, which assume congruence with close relatives. In Proteaceae, no variation is known in monoploid chromosome number within genera, there is very little variation within tribes across 205 species surveyed ([Stace *et al.*, 1998](#_ENREF_44)). Eupolyploidy is rare in the family and there is no indication in our data to suggest eupolyploidy in our species, apart from *Lomatia tasmanica* and *Toronia toru*. The Persoonioid clade included subfamilies Persoonioideae and Bellendenoideae. The Grevilleoid/Proteoid clade included subfamilies Grevilleoideae, Proteoideae and Symphionematoideae. Previously recognised subfamilies Sphalmioideae and Carnarvonioideae are considered here to be part of subfamily Grevilleoideae.

**References**

**Stace HM, Douglas AW, Sampson JF. 1998.** Did ‘paleo-polyploidy’ really occur in Proteaceae? *Australian Systematic Botany* **11:** 613–629.
